# Improving Localization Accuracy under Constrained Regions in Wireless Sensor Networks through Geometry Optimization

**DOI:** 10.3390/e25010032

**Published:** 2022-12-23

**Authors:** Xinpeng Fang, Zhihao He, Shouxu Zhang, Junbing Li, Ranjun Shi

**Affiliations:** 1 School of Aerospace Science and Technology, Xidian University, Xi’an 710071, China; 2 School of Marine Science and Technology, Northwestern Polytechnical University, Xi’an 710072, China; 3 Aeronautical and Astronautical Engineering Collage, Air Force Engineering University, Xi’an 710043, China

**Keywords:** geometry optimization, region constraints, minimum safety distance, D-optimality

## Abstract

In addition to various estimation algorithms, the target localization accuracy in wireless sensor networks (WSNs) can also be improved from the perspective of geometry optimization. Note that existing placement strategies are mainly aimed at unconstrained deployment regions, i.e., the positions of sensors are arbitrary. In this paper, considering factors such as terrain, communication, and security, the optimal range-based sensor geometries under circular deployment region and minimum safety distance constraints are proposed. The geometry optimization problem is modeled as a constrained optimization problem, with a D-optimality-based (maximizing the determinant of FIM matrix) scalar function as the objective function and the irregular feasible deployment regions as the constraints. We transform the constrained optimization problem into an equivalent form using the introduced maximum feasible angle and separation angle, and discuss the optimal geometries based on the relationship between the minimum safety distance and the maximum feasible angle. We first consider optimal geometries for two and three sensors in the localization system, and then use their findings to extend the study to scenarios with arbitrary numbers of sensors and arbitrarily shaped feasible regions. Numerical simulation results are included to verify the theoretical conclusions.

## 1. Introduction

Accurate knowledge of the target position is critical for most potential applications of wireless sensor networks (WSNs), such as remote sensing, indoor positioning, precision agriculture, and military affairs [1,2,3,4]. For range-based localization systems, geographically distributed sensors can obtain multiple noisy measurements by exploiting characteristics of different types of acquired signals, including received signal strength (RSS), time (difference) of arrival (TOA/TDOA), angle of arrival (AOA), and combinations thereof.

Target localization performance can be affected by estimation methods based on the aforementioned measurements, and some effective algorithms have been proposed to improve localization accuracy, such as the well-known weighted least square (WLS) [5,6], maximum likelihood estimator (MLE) [7,8], multidimensional scaling (MDS) [9,10], and Kalman filter (KF) [11,12]. Besides various estimation techniques, another potential factor that has been shown to affect localization performance is the relative positional relationship between the sensors and the target, which can be described by geometric parameters such as distances, azimuths, and shapes [13,14]. This is the geometry optimization problem to be studied in this paper.

The localization problem is essentially an estimation process of the unknown target state, and the Cramer–Rao lower bound (CRLB), which is equal to the inverse of the Fisher information matrix (FIM), can be chosen to characterize the estimation accuracy. Specifically, a symmetric and at least semi-positive definite CRLB or its corresponding FIM matrix can be derived using the geometric parameters, whose positive real eigenvalues are related to the uncertainty ellipsoid. Therefore, we can use some eigenvalue-based derived scalar function as optimization criterion for sensors placement strategies. In this paper, we choose to maximize the determinant of the FIM matrix (i.e., D-optimality) for higher accuracy, which is a commonly used performance standard in geometry optimization research.

Various geometry optimization schemes have been explored in the literature. For instance, the optimal geometries between sensors and target were provided in [15] by minimizing the D-optimality for bearing-only, TOA-based, and range-only localization, respectively. A similar 3D TOA target localization problem was developed in [16], with A-optimality as the evaluation criterion, which is minimizing the trace of the inverse FIM matrix. A unified optimization framework for designing optimal sensor placement was developed in [17] for different type of measurements based on A-, D- and E-optimality (minimizing the maximum eigenvalue of the CRLB matrix) criteria. The recently developed frame theory provides a solution for optimal sensor placement, especially for some complicated scenarios [18,19]. The frame theory was applied in [18] to formulate the optimal anchor placement as an identical parameter optimization problem. Localization accuracy can be further enhanced by optimizing the placement of hybrid sensors compared to using only one type of measurement. The strategy of optimal sensor deployment for static target localization utilizing hybrid RSS-AOA-TOA sensors was developed in [20]. The performance of 3D localization for underwater optical WSNs was analyzed in [21], and a closed form CRLB expression was derived under the presence of uncertainty in anchor node positions. The sufficient and necessary condition for optimal bearing sensor placement was presented in [22], the evaluation function, GDOP, was based on the CRLB, and an efficient algorithm was developed to deploy vehicles to approach the best positions.

However, all the aforementioned studies on optimal geometry have the premise that there are no restrictions on the deployment regions of the sensors. Theoretically, sensors can be placed in the entire space around the target, and subsequently the resulting geometry optimization problem can be treated as unconstrained. In practice, sensors (or UAVs) often face constraints due to terrain, communication or security issues, they cannot simply be placed in arbitrary positions, and their deployment region constraints cannot be ignored. The optimal acoustic sensor placement for multiple underwater target localization was studied in [23], the sensors were all located at the sea surface. Similarly, the sensor AUVs in [24] can only be placed at water surface to investigate the optimal configuration of sensors for RSS-based localization.

This paper aims to study the geometry optimization problem of range-based target localization under certain specific irregular deployment region constraints, expecting to maximize the estimation accuracy. Unlike the existing literature, we impose certain constraints on the feasible deployment region of the sensors with respect to the target, which is similar to [25]. Furthermore, we consider the existence of a minimum safety distance for each sensor-target pair. Specifically, sensors can be placed inside and on the boundary of a circular region, while the target is located outside this region. On the other hand, the distance between each sensor and target cannot be less than the minimum safety distance. Therefore, different minimum safety distances will result in different irregular feasible deployment regions. Under this assumption, the distances and azimuths between the sensors and the target cannot exceed this feasible deployment region, which makes geometry optimization a constrained optimization problem. The contributions of this paper are summarized as follows:

- We consider certain specific irregular deployment region constraints in this paper, which are embodied in two aspects: the initial circular region and the minimum safety distance requirement.
- We describe the optimal geometry as a nonlinear constrained optimization problem. Its objective function is the D-optimality, and the constraints are the irregular feasible positions of the sensors.
- We transform the established constrained optimization problem into an equivalent form expressed by maximum feasible angle and separation angles to reduce the solution complexity.
- We first give the optimal geometries for two and three sensors, respectively, and then extend them to any number of sensors and give some discussions for arbitrarily shaped deployment regions.

The remainder of this paper is organized as follows. We provide the constraints and objective function of the constrained optimization problem and its equivalent form in Section 2. The geometry optimization problems for the localization with two and three sensors are presented in Section 3 and Section 4, respectively. Then, we extend the discussions to the case of any number of sensors and any shaped regions in Section 5. Simulation results are presented in Section 6 to validate the results. Finally, we conclude in Section 7.

## 2. Problem Statement

Under certain deployment region constraints, the range-based target localization problem can be modeled as a constrained optimization problem. In this section, we will give expressions for the irregular feasible deployment region model and the D-optimality-based evaluation criterion, which are, respectively, the constraints and objective function of the obtained constrained optimization problem.

### 2.1. Irregular Feasible Deployment Region

**Figure 1:**
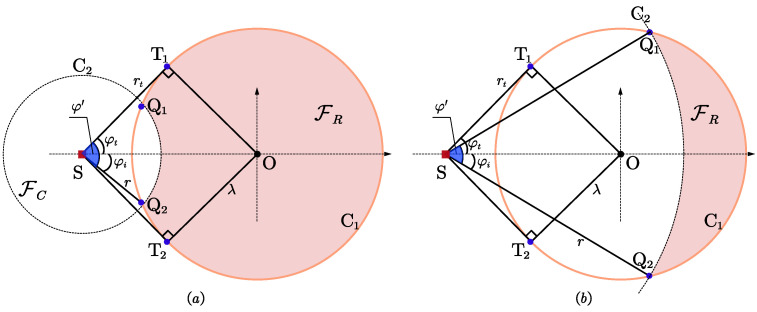
The localization scenario. (**a**) $r\leq r_{t}$; (**b**) $r>r_{t}$.

Let us consider a 2D localization scenario with *n* sensors and one target node, where the positions of sensors, noted as $s_{i}={\left[x_{i},y_{i}\right]}^{T}$, i=1,2,⋯,n, are known a priori, while the position of the target, noted as $s={\left[x_{t},y_{t}\right]}^{T}$, is unknown. Figure 1 shows the localization scenario with the notations indicated.

The considered irregular feasible deployment region of the sensors, $F_{R}$, is determined by two aspects. One is that all sensors can only be placed inside or on the boundary of a circular region, $F_{C}$, while the target is located outside this circular region; the other is that the distance between each sensor and target cannot be less than a certain threshold, i.e., the minimum safety distance *r*. The above constrained scenario is applicable to the situation where the sensors cannot be far away from the target due to communication or measuring distance, and cannot be too close due to collision or concealment factors.

For simplicity and without loss of generality, assume that $C_{1}$ is the boundary of $F_{C}$, whose origin is $O={\left[0,0\right]}^{T}$ and the radius is λ. Let the target be located at $s={\left[-\rho ,0\right]}^{T}$ with ρ>0, $C_{2}$ is the boundary of the circular region with s as the origin and *r* as the radius. Thus, the constraints of the constrained optimization problem can be expressed as the following inequalities.

(1) $$\left\{\begin{array}{c} ∥s∥>\lambda \\ ∥s_{i}∥\leq \lambda \\ ∥s_{i}-s∥\geq r \end{array}\right.i=1,2,\cdots ,n$$

### 2.2. D-Optimality-Based Evaluation Criterion

Collecting all *n* range measurements together, the measurement model in vector form is yields,

(2) $$\hat{r}=r+\varepsilon$$

where $\hat{r}={\left[{\hat{r}}_{1},{\hat{r}}_{2},\cdots ,{\hat{r}}_{n}\right]}^{T}$ is the measurement vector, and $r={\left[r_{1},r_{2},\cdots ,r_{n}\right]}^{T}$ is its actual counterpart with $r_{i}=\sqrt{{\left(x_{i}-x_{t}\right)}^{2}+{\left(y_{i}-y_{t}\right)}^{2}}$. The measurement error vector $\varepsilon ={\left[\varepsilon_{1},\varepsilon_{2},\cdots ,\varepsilon_{N}\right]}^{T}$ is assumed to be zero-mean Gaussian with covariance matrix $Q=\mathrm{diag}\left(\sigma_{1}^{2},\sigma_{2}^{2},\cdots ,\sigma_{n}^{2}\right)\in R^{n\times n}$.

The CRLB expresses a tight bound on the best achievable accuracy of any unbiased estimator of a deterministic parameter, while the entropy can be used to measure the uncertainty of the parameter. In other words, if the entropy of a parameter is high, it means that the parameter is highly uncertain or random, and the CRLB can be used to quantify the precision of an estimator of that parameter. As stated above, the covariance matrix Q is independent of the unknown state s, then the CRLB of s is

(3) $$\mathrm{CRLB}\left(s\right)≜{\left[F\left(s\right)\right]}^{-1}={\left[{\left(\nabla_{s}r\left(s\right)\right)}^{T}Q^{-1}\left(\nabla_{s}r\left(s\right)\right)\right]}^{-1}$$

where $F\left(s\right)$ denotes the corresponding FIM, and $\nabla_{s}r\left(s\right)$ is the Jacobian matrix of r with respect to s, which is stated as

(4) $$\nabla_{s}r\left(s\right)={\left[\begin{array}{ccc} \frac{\partial r_{1}}{\partial s_{1}} & \cdots & \frac{\partial r_{N}}{\partial s_{2}}\\ \frac{\partial r_{1}}{\partial s_{2}} & \cdots & \frac{\partial r_{N}}{\partial s_{2}} \end{array}\right]}^{T}$$

Since the unknown target state $s={\left[x_{t},y_{t}\right]}^{T}$, then we have $\partial r_{1}/\partial s_{1}=\left(x_{i}-x_{t}\right)/r_{i}=\Delta x_{i}/r_{i}$, $\partial r_{1}/\partial s_{2}=\left(y_{i}-y_{t}\right)/r_{i}=\Delta y_{i}/r_{i}$. After some algebraic manipulations, the full symmetric FIM expression is obtained as

(5) $$F\left(s\right)=\sum_{i=1}^{n}\left[\begin{array}{cc} \frac{\Delta {x_{i}}^{2}}{\sigma_{i}^{2}r_{i}^{2}} & \frac{\Delta x_{i}\Delta y_{i}}{\sigma_{i}^{2}r_{i}^{2}}\\ \frac{\Delta x_{i}\Delta y_{i}}{\sigma_{i}^{2}r_{i}^{2}} & \frac{\Delta {y_{i}}^{2}}{\sigma_{i}^{2}r_{i}^{2}} \end{array}\right]$$

The FIM characterizes the amount of information contained in the measurement vector r used to estimate s, and more information implies a more accurate position estimation. Since the eigenvalues of the CRLB matrix construct the uncertainty ellipsoid in the position estimation, the D-optimality is to minimize the volume of the uncertainty ellipsoid, i.e., the objective function of the constrained optimization problem is

(6) $$\det F\left(s\right)=\sum_{i=1}^{n}\frac{\Delta {x_{i}}^{2}}{\sigma_{i}^{2}r_{i}^{2}}\sum_{i=1}^{n}\frac{\Delta {y_{i}}^{2}}{\sigma_{i}^{2}r_{i}^{2}}-{\left(\sum_{i=1}^{n}\frac{\Delta x_{i}\Delta y_{i}}{\sigma_{i}^{2}r_{i}^{2}}\right)}^{2}$$

### 2.3. Equivalent Constrained Optimization Problem

Due to the irregular feasible deployment region constraints of sensors in the localization system, the constrained optimization problem for geometry optimization can be established by combining (1) and (6) as follows.

(7) $$\begin{array}{c} \max\det F\left(s\right)\\ s.t.\left\{\begin{array}{c} ∥s∥>\lambda \\ ∥s_{i}∥\leq \lambda \\ ∥s_{i}-s∥\geq r \end{array}\right. \end{array}$$

To solve the above constrained optimization problem, we introduce the maximum feasible angle and separation angle to transform (7) into an equivalent form. As depicted in Figure 1, the two intersections of $C_{1}$ and $C_{2}$ are denoted as $Q_{1}$ and $Q_{2}$, whose azimuth relative to the target is $\varphi_{i}$, the two tangent points of the target with respect to $C_{1}$ are denoted as $T_{1}$ and $T_{2}$, the corresponding tangent angle is $\varphi_{t}$, the tangent line is $r_{t}$, then we obtain $r_{t}=\sqrt{\rho^{2}-\lambda^{2}}$, $\varphi_{t}<\pi /2$, $\varphi_{i}\leq \varphi_{t}$.

Since all sensors and target can only be deployed in the regions determined by (1), we define the maximum feasible angle, $\varphi^{\prime }$, as the maximum field of view of the target with respect to the region $F_{R}$, i.e., the $∠T_{1}{\mathrm{ST}}_{2}$ and $∠Q_{1}{\mathrm{SQ}}_{2}$ in Figure 1a,b, respectively. We can conclude that different minimum safety distances will result in different shapes of irregular feasible regions and different maximum feasible angles.

- If r>ρ+λ, $F_{R}$ is an empty set, $F_{R}=⌀$.
- If $r_{t}<r\leq \rho +\lambda$, $F_{R}$ is a crescent-shaped region, and its maximum feasible angle is $\varphi^{\prime }=2\varphi_{i}$, $\varphi_{i}<\varphi_{t}$.
- If $\rho -\lambda <r\leq r_{t}$, $F_{R}$ is a crescent-shaped region, and its maximum feasible angle is $\varphi^{\prime }=2\varphi_{t}$.
- If r≤ρ−λ, the minimum safety distance has no effect on $F_{C}$, i.e., $F_{R}=F_{C}$, the maximum feasible angle is $\varphi^{\prime }=2\varphi_{t}$.

Denote $\theta_{i}$ as the azimuth of the *i*th sensor with respect to the target, it follows that $\Delta x_{i}/r_{i}=\cos\theta_{i}$, $\Delta y_{i}/r_{i}=\sin\theta_{i}$. The *separation angle*, $\theta_{ij}$, is defined as the angle of the *i*th and *j*th sensors subtended at the target, $\theta_{ij}\leq \pi$. As stated above, if r>ρ+λ or r≤ρ−λ, we have $F_{R}=⌀$ and $F_{R}=F_{C}$, respectively. In this paper, we mainly focus on the irregular feasible deployment regions, i.e., ρ−λ<r≤ρ+λ.

Applying the maximum feasible angle and the separation angle, the constrained optimization problem (7) can be transformed into the following equivalent form.

(8) $$\begin{array}{c} \max\sum_{i=1}^{n}\sum_{i<j}^{n}\sigma_{i}^{-2}\sigma_{j}^{-2}\sin^{2}\theta_{ij}\\ s.t.\left\{\begin{array}{cc} \theta_{ij}\leq 2\varphi_{t},\varphi_{t}<\pi /2 & \mathrm{if}\;\rho -\lambda <r\leq r_{t}\\ \theta_{ij}\leq 2\varphi_{i},\varphi_{i}<\varphi_{t}<\pi /2 & \mathrm{if}\;r_{t}<r\leq \rho +\lambda \\ ∥s_{i}-s∥\geq r & \end{array}\right. \end{array}$$

**Remark** **1.** *A more realistic scenario of (8) is that different sensors have different noise variances $\sigma_{i}$, however, it is quite difficult or even impossible to find its exact closed-form solutions, especially for n≥4. For this reason, the special case of n sensors with the same variance is often considered in geometry optimization or practical applications [22,26,27]. This paper is also conducted under this assumption, i.e., $\sigma_{1}=\sigma_{2}=\cdots =\sigma_{n}=\sigma$.*

Under the assumption in Remark 1, the objective function expression in (8) is just a mathematical operation of different separation angles, while the last distances constraint is independent of the separation angles. Therefore, we can first solve the optimization problem while ignoring the last constraint, and then consider the effect of the minimum safety distance separately.

For the case of a large number of sensors, the grouping method in [28] is a convenient tool to give a more comprehensive optimal geometries, its main idea is to divide the sensors into subgroups containing two or three sensors and guarantee that each subgroup has an optimal geometry. The corresponding optimal geometries for n=2 and n=3 are summarized in the following theorem.

**Theorem** **1** ([15]). *For the range-based localization system without any deployment region constraints, the evaluation function is the same as (6). The sensors for n=2 should be placed with the separation angle of $\theta_{12}=\pi /2$, while the sensors for n=3 should be placed with the combination of separation angles of $\left\{\theta_{13},\theta_{23},\theta_{12}\right\}=\left\{\pi /3,\pi /3,2\pi /3\right\}$ or $\left\{2\pi /3,2\pi /3,2\pi /3\right\}$. The distance between each sensor and target can be arbitrary.*

## 3. Geometry Optimization for Two Sensors

We can conclude from Theorem 1 that if there are no restrictions on the deployment regions of the sensors, then they can be placed around the target, and the optimal separation angle is $\theta^{*}=\pi /2$. However, since the target is located outside $F_{C}$, which is equivalent to the sensors being all placed on the same side of the target, the maximum feasible angle that can be obtained may be smaller than this optimal separation angle, i.e., $\varphi^{\prime }<\pi /2$. Therefore, we can draw a comprehensive conclusion on the geometry optimization by comparing the maximum feasible angle and the optimal separation angle in the following. The relationship between distance parameters and maximum feasible angle is summarized in Table 1, where $r_{1}$ is described in Section 3.2.

### 3.1. The Tangent Angle $\varphi_{t}\leq \pi /4$

The tangent angle $\varphi_{t}\leq \pi /4$ means that $\rho \geq \sqrt{2}\lambda$, $r_{t}\geq \lambda$, the geometry optimization problem can be stated as the following (9). It can be seen that the separation angle $\theta_{12}\leq \pi /2$ always holds, then it is easy to prove that the objective function is a monotonically increasing function on $\theta_{12}$ and the separation angle should be as large as possible.

(9) $$\begin{array}{c} \max\sigma^{-4}\sin^{2}\theta_{12}\\ s.t.\left\{\begin{array}{cc} \theta_{12}\leq \varphi^{\prime }=2\varphi_{t},\varphi_{t}\leq \pi /4 & \rho -\lambda <r\leq r_{t}\\ \theta_{12}\leq \varphi^{\prime }=2\varphi_{i},\varphi_{i}<\varphi_{t}\leq \pi /4 & r_{t}<r\leq \rho +\lambda \\ r_{1}\geq r,r_{2}\geq r \end{array}\right. \end{array}$$

Specifically, if $\rho -\lambda <r\leq r_{t}$, we have $\theta_{12}\leq 2\varphi_{t}$, the objective function gets its maximum at $\theta_{12}=2\varphi_{t}$, two sensors should be placed at two tangent points $T_{1}$ and $T_{2}$, respectively, which are symmetrical about the *x*-axis. The maximum of the objective function is $\sigma^{-4}\sin^{2}2\varphi_{t}$, and the coordinates of two sensors are $S_{1}\left(-\lambda^{2}/\rho ,\lambda r_{t}/\rho \right)$, $S_{2}\left(-\lambda^{2}/\rho ,-\lambda r_{t}/\rho \right)$. Similarly, if $r_{t}<r\leq \rho +\lambda$, we still have $\theta_{12}\leq 2\varphi_{i}<\pi /2$, we can obtain the conclusion that the two sensors should be placed at two intersections $Q_{1}$ and $Q_{2}$, respectively, with the maximum of $\sigma^{-4}\sin^{2}2\varphi_{i}$. The coordinates of two sensors are $S_{1}\left(r\cos\varphi_{i}-\rho ,r\sin\varphi_{i}\right)$, $S_{2}\left(r\cos\varphi_{i}-\rho ,-r\sin\varphi_{i}\right)$, where $\varphi_{i}=\arccos\left(\left(r^{2}+r_{t}^{2}\right)/\left(2r\rho \right)\right)$.

**Figure 2:**
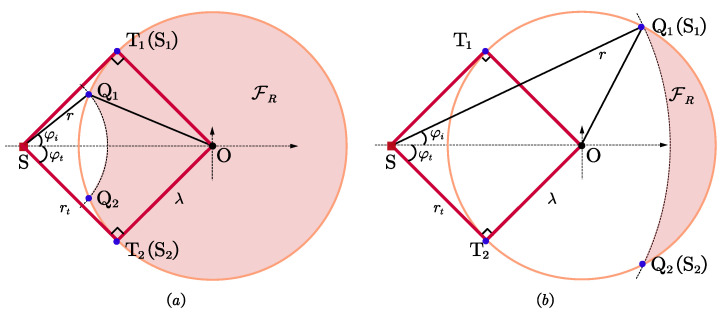
Optimal geometries for n=2, $\varphi_{t}\leq \pi /4$. (**a**) $\rho -\lambda <r\leq r_{t}$; (**b**) $r_{t}<r\leq \rho +\lambda$.

The optimal geometries for n=2, $\varphi_{t}\leq \pi /4$ are presented in Figure 2, there is only one optimal geometry for each of the above two cases.

### 3.2. The Tangent Angle $\varphi_{t}>\pi /4$

**Figure 3:**
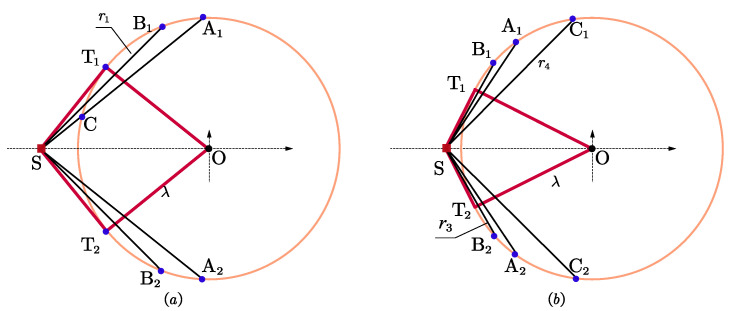
Illustration of parameters. (**a**) n=2, $\varphi_{t}>\pi /4$; (**b**) n=3, $\varphi_{t}>\pi /3$.

For $r_{t}<\lambda$, the tangent angle $\varphi_{t}$ will be greater than π/4. As depicted in Figure 3a, suppose $∠T_{1}{\mathrm{SA}}_{2}=∠T_{2}{\mathrm{SA}}_{1}=\pi /2$, $∠B_{1}\mathrm{SO}=∠B_{2}\mathrm{SO}=\pi /4$, the lengths of ${\mathrm{SB}}_{1}$ is marked by $r_{1}$. If the slope of ${\mathrm{ST}}_{1}$ is *k*, then the slopes of ${\mathrm{ST}}_{2}$, ${\mathrm{SA}}_{1}$ and ${\mathrm{SA}}_{2}$ are −k, 1/k, and −1/k, respectively, where $k=\lambda /r_{t}$. This gives $r_{1}=\left(\rho +\sqrt{\lambda^{2}-r_{t}^{2}}\right)/\sqrt{2}$.

**Figure 4:**
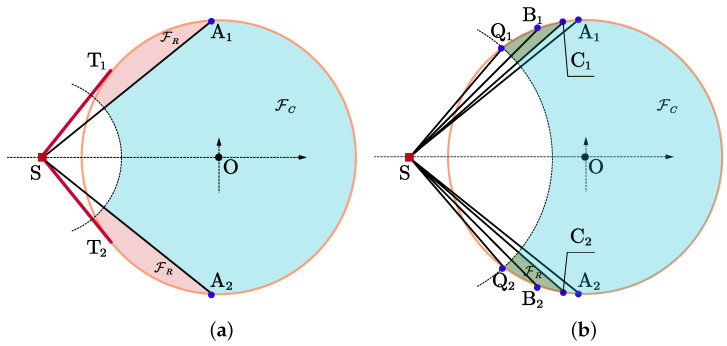
Optimal geometries for n=2, $\varphi_{t}\geq \pi /4$, $r\leq r_{1}$. (**a**) $\rho -\lambda <r\leq r_{t}$; (**b**) $r_{t}<r\leq r_{1}$.

If $\rho -\lambda <r\leq r_{1}$, the maximum feasible angle is greater than π/2, we just need to ensure that two sensors are placed with $\theta_{12}=\pi /2$ to achieve the optimal geometry, and the maximum is $\sigma^{-4}$. It is worth noting that the difference between $\rho -\lambda <r\leq r_{t}$ and $r_{t}<r\leq r_{1}$ lies in the maximum feasible angle, which is $2\varphi_{t}$ and $2\varphi_{i}$, respectively. The irregular feasible deployment regions $F_{R}$ are presented in Figure 4. Although $F_{C}$ satisfies the constraints (1), the separation angle $\theta_{12}$ cannot reach π/2 as long as one sensor is located in this region, so $F_{C}$ is actually an infeasible region, where $∠Q_{1}{\mathrm{SC}}_{2}=∠Q_{2}{\mathrm{SC}}_{1}=\pi /2$ in Figure 4b. If $r_{1}<r\leq \rho +\lambda$, $\varphi^{\prime }$ will be less than π/2, as analyzed in Section 3.1, the two sensors should be positioned so that the separation angle is maximized, i.e., they should be placed at two intersections with a maximum of $\sigma^{-4}\sin^{2}2\varphi_{i}$.

In a word, if the maximum feasible angle is greater than π/2, the two sensors should be placed in such a way as to form the separation angle π/2; otherwise, the two sensors should be placed with the highest possible separation angle.

## 4. Geometry Optimization for Three Sensors

Consider the case where three sensors are located in a circular region with the same constraints as in Section 3. According to the Theorem 1, the optimal combination of separation angles, $\left\{\pi /3,\pi /3,2\pi /3\right\}$, can be obtained as long as the maximum feasible angle is not less than 2π/3, furthermore, if the maximum feasible angle is greater than or equal to 4π/3, the optimal combination of $\left\{2\pi /3,2\pi /3,2\pi /3\right\}$ can also be achieved. However, since the target lies outside $F_{C}$, the maximum feasible angle is affected by several distance parameters, and the specific relationship is shown in Table 2.

Since the parameter σ can be regarded as the scale coefficient of the objective function, it is assumed that σ=1 in the subsequent analysis. In addition, the third sensor $S_{3}$ is assumed to be located between the other two sensors, $S_{1}$ and $S_{2}$, i.e., $\theta_{12}=\theta_{13}+\theta_{23}$.

### 4.1. The Tangent Angle $\varphi_{t}\leq \pi /4$

If the tangent angle $\varphi_{t}\leq \pi /4$, we have $\rho \geq \sqrt{2}\lambda$, $r_{t}\geq \lambda$. For a smaller minimum safety distance, $r\leq r_{t}$, the objective function becomes $\Psi_{0}=\sin^{2}\theta_{13}+\sin^{2}\theta_{12}+\sin^{2}\theta_{23}$ with $\theta_{12},\theta_{13},\theta_{23}\leq 2\varphi_{t}\leq \pi /2$. Taking its derivative with respect to $\theta_{12}$, gives

(10) $$\frac{\partial \Psi_{0}}{\partial \theta_{12}}=2\sin\left(2\theta_{12}-\theta_{23}\right)\cos\theta_{23}=2\sin\left(\theta_{12}+\theta_{13}\right)\cos\theta_{23}$$

Note that $\theta_{12},\theta_{13},\theta_{23}\leq \pi /2$, and thus, $\theta_{12}+\theta_{13}\leq \pi$, $\sin\left(\theta_{12}+\theta_{13}\right)>0$, $\cos\theta_{23}>0$. As a result, the objective function increases with $\theta_{12}$, its maximum is obtained at $\theta_{12}=2\varphi_{t}$, then the objective function becomes $\Psi_{1}=\sin^{2}2\varphi_{t}+\sin^{2}\left(2\varphi_{t}-\theta_{23}\right)+\sin^{2}\theta_{23}$, its derivative with respect to $\theta_{23}$ is $\partial \Psi_{1}/\partial \theta_{23}=2\sin2\left(\theta_{23}-\varphi_{t}\right)\cos2\varphi_{t}$ with $\cos2\varphi_{t}>0$. It is easy to deduce that $\Psi_{1}$ gets its maximum, $2\sin^{2}2\varphi_{t}$, at $\theta_{23}=0$ or $2\varphi_{t}$, which means that the optimal solutions are $\left\{\theta_{13},\theta_{23},\theta_{12}\right\}=\left\{2\varphi_{t},0,2\varphi_{t}\right\}$ or $\left\{0,2\varphi_{t},2\varphi_{t}\right\}$, i.e., the optimal geometry is to place sensors $S_{1}$ and $S_{2}$ at tangent points $T_{1}$ and $T_{2}$, respectively, and $S_{3}$ is randomly placed at any tangent point with the maximum of $2\sin^{2}2\varphi_{t}$, the coordinates of three sensors are $S_{1}\left(-\lambda^{2}/\rho ,\lambda r_{t}/\rho \right)$, $S_{2}\left(-\lambda^{2}/\rho ,-\lambda r_{t}/\rho \right)$, $S_{3}\left(-\lambda^{2}/\rho ,\pm \lambda r_{t}/\rho \right)$.

When the minimum safety distance satisfies $r_{t}<r\leq \rho +\lambda$, the maximum separation angle will be $\theta_{12},\theta_{13},\theta_{23}\leq 2\varphi_{i}<\pi /2$, then we arrive at $\theta_{12}+\theta_{13}\leq 4\varphi_{i}<\pi$, $\theta_{23}\leq 2\varphi_{i}<\pi /2$, $\sin\left(\theta_{12}+\theta_{13}\right)>0$, $\cos\theta_{23}>0$, the optimal geometries can be achieved by placing $S_{1}$ and $S_{2}$ at intersections $Q_{1}$ and $Q_{2}$, respectively, and placing the third sensor $S_{3}$ at either intersection with a maximum of $2\sin^{2}2\varphi_{i}$. The coordinates of the sensors are $S_{1}\left(r\cos\varphi_{i}-\rho ,r\sin\varphi_{i}\right)$, $S_{2}\left(r\cos\varphi_{i}-\rho ,-r\sin\varphi_{i}\right)$, $S_{3}\left(r\cos\varphi_{i}-\rho ,\pm r\sin\varphi_{i}\right)$.

### 4.2. The Tangent Angle $\pi /4<\varphi_{t}\leq \pi /3$

If the tangent angle $\varphi_{t}=\pi /3$, we have $\rho =2\sqrt{3}\lambda /3$, $r_{t}=\sqrt{3}\lambda /3$. Hence, $\pi /4<\varphi_{t}\leq \pi /3$ means that $2\sqrt{3}\lambda /3\leq \rho <\sqrt{2}\lambda$, $\sqrt{3}\lambda /3\leq r_{t}<\lambda$. For the minimum safety distance $r\leq r_{t}$, the separation angles satisfy $\theta_{12},\theta_{13},\theta_{23}\leq \varphi^{\prime }=2\varphi_{t}\in \left(\pi /2,2\pi /3\right]$. The derivative of objective function with respect to $\theta_{13}$ is

(11) $$\frac{\partial \Psi_{0}}{\partial \theta_{13}}=2\sin\left(2\theta_{13}-\theta_{12}\right)\cos\theta_{12}=2\sin\left(\theta_{13}-\theta_{23}\right)\cos\theta_{12}$$

After analysis, it is obvious that for $\theta_{12}\leq \pi /2$, there exists $\cos\theta_{12}\geq 0$, the derivative changes from negative to positive as $\theta_{13}$ increases, implying that $\Psi_{0}$ is minimum at $\theta_{13}=\theta_{23}$ and maximum at $\theta_{13}=0$ or $\theta_{12}$, the maximum is $2\sin^{2}\theta_{12}$. Since $\theta_{12}$ can definitely take π/2, then the maximum for $\theta_{12}\leq \pi /2$ is 2, which is obtained at $\theta_{12}=\pi /2$, $\theta_{13}=0$ or π/2. On the other hand, if $\pi /2<\theta_{12}\leq 2\pi /3$, then $\cos\theta_{12}<0$, and $\Psi_{0}$ achieves its maximum at $\theta_{13}=\theta_{23}=\theta_{12}/2$, the maximum is $\Psi_{2}=\sin^{2}\theta_{12}+2\sin^{2}\left(\theta_{12}/2\right)$. Its derivative with respect to $\theta_{12}$ is $\partial \Psi_{2}/\partial \theta_{12}=\sin2\theta_{12}+\sin\theta_{12}=2\sin\left(3\theta_{12}/2\right)\cos\left(\theta_{12}/2\right)$. It follows from $\theta_{12}\in \left[0,2\pi /3\right]$ that $\sin\left(3\theta_{12}/2\right)>0$ and $\cos\left(\theta_{12}/2\right)>0$, then $\Psi_{2}$ gets its maximum at $\theta_{12}=2\varphi_{t}$, that is, the maximum for $\pi /2<\theta_{12}\leq 2\pi /3$ is $\sin^{2}2\varphi_{t}+2\sin^{2}\varphi_{t}$, which is obtained at $\theta_{13}=\theta_{23}=\theta_{12}/2=\varphi_{t}$.

**Figure 5:**
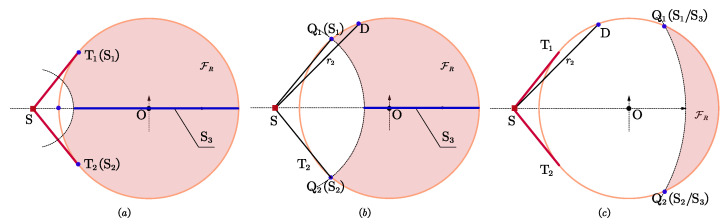
Optimal geometries for n=3, $\pi /4<\varphi_{t}\leq \pi /3$. (**a**) $r\leq r_{t}$; (**b**) $r_{t}<r\leq r_{2}$; (**c**) $r>r_{2}$.

The final maximum for $r\leq r_{t}$ needs to be obtained by comparing the above two maxima. It can be seen that $2-\left(\sin^{2}2\varphi_{t}+2\sin^{2}\varphi_{t}\right)=\cos^{2}2\varphi_{t}+\cos2\varphi_{t}<0$ holds for arbitrary $\varphi_{t}$, then the maximum is achieved at $\theta_{12}=2\varphi_{t}$, $\theta_{13}=\theta_{23}=\varphi_{t}$, the maximum is $\sin^{2}2\varphi_{t}+2\sin^{2}\varphi_{t}$. That is $S_{1}$ and $S_{2}$ are placed at $T_{1}$ and $T_{2}$, respectively, $S_{3}$ is placed at the intersections of the bisector of $∠T_{1}{\mathrm{ST}}_{2}$ and $F_{R}$, as shown in Figure 5a, the coordinates are $S_{1}\left(-\lambda^{2}/\rho ,\lambda r_{t}/\rho \right)$, $S_{2}\left(-\lambda^{2}/\rho ,-\lambda r_{t}/\rho \right)$, $S_{3}\left(x_{3},0\right)$, where $x_{3}\in \left[r-\rho ,\lambda \right]$.

**Remark** **2.** *The above derivation yields that for the unconstrained case, the optimal solution is still $\theta_{12}=2\varphi_{t}$, $\theta_{13}=\theta_{23}=\varphi_{t}$, and two sensors should be placed at two tangent points, respectively, a third sensor should be placed at the intersections of the bisector of $∠T_{1}{\mathrm{ST}}_{2}$ and $F_{C}$.*

Denote the length of SD in Figure 5b is $r_{2}$, ∠DSO=π/4, yields $r_{3}=\left(\rho +\sqrt{\lambda^{2}-r_{t}^{2}}\right)/\sqrt{2}$. If the minimum safety distance $r_{t}<r\leq r_{2}$, we have the separation angles $\theta_{12},\theta_{13},\theta_{23}\leq \varphi^{\prime }=2\varphi_{i}\in \left(\pi /2,2\varphi_{t}\right)$, $2\varphi_{t}\leq 2\pi /3$. In this case, the analysis process and conclusion are similar to those in $r\leq r_{t}$. We obtain the optimal geometries with two sensors placed at two intersections $Q_{1}$ and $Q_{2}$ and a third sensor placed at the intersections of the bisector of $∠Q_{1}{\mathrm{SQ}}_{2}$ and $F_{R}$. The maximum is $2\sin^{2}\varphi_{i}+\sin^{2}2\varphi_{i}$, and the coordinates of the three sensors are $S_{1}\left(\rho -r\cos\varphi_{i},r\sin\varphi_{i}\right)$, $S_{2}\left(\rho -r\cos\varphi_{i},-r\sin\varphi_{i}\right)$, $S_{3}\left(x_{3},0\right)$.

When the minimum safety distance is greater than $r_{2}$, the maximum feasible angle $\varphi^{\prime }\leq 2\varphi_{i}<\pi /2$ holds, which is the same as that in Section 4.1, the objective function gets its maximum at $\theta_{12}=2\varphi_{i}$, $\theta_{13}=0$ or $2\varphi_{i}$, the maximum is $2\sin^{2}2\varphi_{i}$. As shown in Figure 5c, two sensors are placed at intersections $Q_{1}$ and $Q_{2}$, respectively, and a third sensor is placed at either intersection.

### 4.3. The Tangent Angle φ>π/3

Denote the lengths of ${\mathrm{SB}}_{1}$ and ${\mathrm{SC}}_{1}$ in Figure 3b as $r_{3}$ and $r_{4}$, respectively, and $∠T_{1}{\mathrm{SA}}_{2}=∠T_{2}{\mathrm{SA}}_{1}=2\pi /3$, $∠B_{1}\mathrm{SO}=∠B_{2}\mathrm{SO}=\pi /3$, $∠C_{1}\mathrm{SO}=∠C_{2}\mathrm{SO}=\pi /4$. After some straightforward manipulations, we can obtain that $r_{3}=\left(\rho +\sqrt{4\lambda^{2}-3\rho^{2}}\right)/2$, $r_{4}=\left(\rho +\sqrt{2\lambda^{2}-\rho^{2}}\right)/\sqrt{2}$.

If $r\leq r_{3}$, we have the separation angles $\theta_{12},\theta_{13},\theta_{23}\leq \varphi^{\prime }>2\pi /3$, the combination of separation angles {π/3,π/3,2π/3} can be achieved, the maximum is 9/4. The difference between $r\leq r_{t}$ and $r_{t}<r\leq r_{3}$ just lies in the maximum feasible angle, which is $2\varphi_{t}$ and $2\varphi_{i}$, respectively, the conclusions are the same. If $r_{3}<r\leq r_{4}$, the maximum feasible angle is $\varphi^{\prime }\in \left(\pi /2,2\pi /3\right]$. Applying the conclusions in Section 4.2 gives the optimal solution as $\theta_{12}=2\varphi_{i}$, $\theta_{13}=\varphi_{i}$, the maximum is $2\sin^{2}\varphi_{i}+\sin^{2}2\varphi_{i}$. Two sensors should be placed at interactions $Q_{1}$ and $Q_{2}$, respectively, a third sensor should be located at the interactions of the bisector of $∠C_{1}{\mathrm{SC}}_{2}$ and $F_{R}$.

For the case of $r>r_{4}$, the maximum feasible angle $\varphi^{\prime }$ is less than π/2, as stated in Section 4.1, the objective function gets its maximum, $2\sin^{2}2\varphi_{i}$, at $\theta_{12}=2\varphi_{i}$, $\theta_{13}=0$ or $2\varphi_{i}$. In order to achieve the optimal separation angles, we can place two sensors at interactions $Q_{1}$ and $Q_{2}$, respectively, and a third sensor at either interaction.

## 5. Extension to Arbitrary Numbers and Shapes

For the geometry optimization problem with n≥ four sensors, we draw on the grouping method to try to divide the sensors into subgroups containing two or three sensors. It should be noted that for some cases, there may be infinitely many optimal geometries, and we only give some sufficient condition.

### 5.1. The Tangent Angle $\varphi_{t}>\pi /3$

As mentioned previously, if the maximum feasible angle $\varphi^{\prime }\geq \pi /2$, the optimal separation angle π/2 can be achieved for the subgroups with two sensors, while if $\varphi^{\prime }\geq 2\pi /3$, the combination of separation angles $\left\{\pi /3,\pi /3,2\pi /3\right\}$ can be achieved for the subgroups with three sensors.

Therefore, if $r\leq r_{3}$, we have $\varphi^{\prime }\geq 2\pi /3$, we can divide the sensors into subgroups with two or three sensors, and guarantee that the separation angle for the subgroups with two sensors is π/2, the separation angles for the subgroups with three sensors is $\left\{\pi /3,\pi /3,2\pi /3\right\}$. For example, if there are twelve sensors in the localization system, n=12, we can divide them into six subgroups containing two sensors, $\left\{2,2,2,2,2,2\right\}$, or into four subgroups containing three sensors, $\left\{3,3,3,3\right\}$, or into mixed subgroups $\left\{2,2,2,3,3\right\}$.

For the case of $r_{3}<r\leq r_{4}$, the maximum feasible angle $\varphi^{\prime }\in \left[\pi /2,2\pi /3\right)$. If the number of the sensors is even, n=2 m, $m\in N^{+}$, we can divide the sensors in pairs into *m* subgroups with separation angle of π/2. However, simply grouping an odd number of sensors may not guarantee overall optimization. Rearranging the objective function, we have

(12) $$\begin{array}{cc} \sum_{i=1}^{n}\sum_{i<j}^{n}\sin^{2}\theta_{ij} & =\sum_{i=1}^{n}\sin^{2}\theta_{i}\sum_{i=1}^{n}\cos^{2}\theta_{i}-\frac{1}{4}{\left(\sum_{i=1}^{n}\sin2\theta_{i}\right)}^{2}\\ \; & =\Upsilon_{1}-\frac{1}{4}{\left(\sum_{i=1}^{n-1}\sin2\theta_{i}\right)}^{2}-\frac{1}{2}\sin2\theta_{n}\sum_{i=1}^{n-1}\sin2\theta_{i}\leq \Upsilon_{1} \end{array}$$

where $\Upsilon_{1}=\sin^{2}\theta_{n}\sum_{i=1}^{n-1}\cos^{2}\theta_{i}+\cos^{2}\theta_{n}\sum_{i=1}^{n-1}\sin^{2}\theta_{i}+\sum_{i=1}^{n-1}\sin^{2}\theta_{i}\sum_{i=1}^{n-1}\cos^{2}\theta_{i}$, and the equality holds when $\sum_{i=1}^{n-1}\sin2\theta_{i}=0$. That is to say, as long as we can make n−1 sensors satisfy $\sum_{i=1}^{n-1}\sin2\theta_{i}=0$, the maximum of the objective function can be achieved.

To this end, we can divide these n−1 sensors into (n−1)/2 subgroups with two sensors and ensure that the two sensors in each subgroup are symmetric about the *x*-axis, then $\theta_{j}+\theta_{j+1}=0$, and $\sin2\theta_{j}+\sin2\theta_{j+1}=0$. For the maximum

(13) $$\begin{array}{cc} \Upsilon_{1} & =\sin^{2}\theta_{n}\sum_{i=1}^{n-1}\cos^{2}\theta_{i}+\cos^{2}\theta_{n}\sum_{i=1}^{n-1}\sin^{2}\theta_{i}+\sum_{i=1}^{n-1}\sin^{2}\theta_{i}\sum_{i=1}^{n-1}\cos^{2}\theta_{i}\\ \; & =\left(n-1-\Upsilon_{11}\right)\sin^{2}\theta_{n}+\Upsilon_{11}\cos^{2}\theta_{n}+\Upsilon_{11}\left(n-1-\Upsilon_{11}\right)\\ \; & =-\Upsilon_{11}^{2}+\left(n-2\sin^{2}\theta_{n}\right)\Upsilon_{11}+\left(n-1\right)\sin^{2}\theta_{n} \end{array}$$

where $\Upsilon_{11}=\sum_{i=1}^{n-1}\sin^{2}\theta_{i}$. By setting to zero the derivative of $\Upsilon_{1}$ with respect to $\Upsilon_{11}$,

(14) $$\frac{\partial \Upsilon_{1}}{\partial \Upsilon_{11}}=-2\Upsilon_{11}+\left(n-2\sin^{2}\theta_{n}\right)=0$$

then $\sum_{i=1}^{n-1}\sin^{2}\theta_{i}=n/2-\sin^{2}\theta_{n}$, i.e., $\sum_{i=1}^{n}\sin^{2}\theta_{i}=n/2$. This allows us to give an sufficient condition to satisfy the optimal geometries as follows:

(15) $$\left\{\begin{array}{c} \sum_{i=1}^{n-1}\sin2\theta_{i}=0\\ \sum_{i=1}^{n}\sin^{2}\theta_{i}=n/2 \end{array}\right.$$

Substituting (15) into (13) leads to $\Upsilon_{1}=\sum_{i=1}^{n}\sin^{2}\theta_{i}\sum_{i=1}^{n}\cos^{2}\theta_{i}-\sin^{2}\theta_{n}\cos^{2}\theta_{n}=\left(n^{2}-\sin^{2}2\theta_{n}\right)/4$, then the maximum of $\Upsilon_{1}$, $n^{2}/4$, is obtained at $\sin2\theta_{n}=0$. It follows from $r_{3}<r\leq r_{4}$ that $\varphi_{i}<\pi /3$, this gives $\theta_{n}=0$, the *n*th sensor is located at the interactions of the bisector of $∠C_{1}{\mathrm{SC}}_{2}$ and $F_{R}$. Then, the sufficient condition (15) becomes

(16) $$\left\{\begin{array}{c} \sum_{i=1}^{n-1}\sin2\theta_{i}=0\\ \sum_{i=1}^{n-1}\sin^{2}\theta_{i}=n/2 \end{array}\right.$$

**Figure 6:**
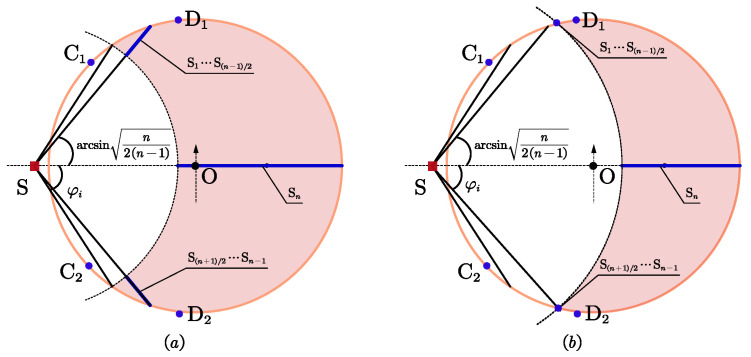
Optimal geometries for *n* is odd, $\varphi_{t}>\pi /3$, $r_{3}<r\leq r_{4}$. (**a**) $\varphi_{i}\geq \mathrm{arc}\sin\sqrt{n/(2n-2)}$; (**b**) $\varphi_{i}<\mathrm{arc}\sin\sqrt{n/(2n-2)}$.

As shown in Figure 6a, if $\varphi_{i}\geq \mathrm{arc}\sin\sqrt{n/(2n-2)}$, a possible sufficient solution is

(17) $$\left\{\begin{array}{cc} \theta_{i}=\mathrm{arc}\sin\sqrt{n/(2n-2)} & i=1,\cdots ,\left(n-1\right)/2\\ \theta_{i}=-\mathrm{arc}\sin\sqrt{n/(2n-2)} & i=\left(n+1\right)/2,\cdots ,n-1\\ \theta_{i}=0 & i=n \end{array}\right.$$

Otherwise, we arrive at $\sum_{i=1}^{n-1}\sin^{2}\theta_{i}<n/2$. Substituting it into (14), leads to $\partial \Upsilon_{1}/\partial \Upsilon_{11}>0$.

Given that $\theta_{i}\leq \varphi_{i}<\mathrm{arc}\sin\sqrt{n/(2n-2)}\leq \pi /3$, in order to maximize $\Upsilon_{1}$, $\sin^{2}\theta_{i}$ should be as large as possible, as shown in Figure 6b, a possible sufficient solution is

(18) $$\left\{\begin{array}{cc} \theta_{i}=\varphi_{i} & i=1,\cdots ,\left(n-1\right)/2\\ \theta_{i}=-\varphi_{i} & i=\left(n+1\right)/2,\cdots ,n-1\\ \theta_{i}=0 & i=n \end{array}\right.$$

this means that (n−1)/2 sensors are placed at each of the two intersections.

For the case of $r>r_{4}$, the maximum feasible angle is $\varphi^{\prime }<\pi /2$. Similarly, if *n* is even, we rearrange the objective function, yields

(19) $$\sum_{i=1}^{n}\sum_{i<j}^{n}\sin^{2}\theta_{ij}=\sum_{i=1}^{n}\sin^{2}\theta_{i}\sum_{i=1}^{n}\cos^{2}\theta_{i}-\frac{1}{4}{\left(\sum_{i=1}^{n}\sin2\theta_{i}\right)}^{2}\leq \Upsilon_{2}$$

where $\Upsilon_{2}=\sum_{i=1}^{n}\sin^{2}\theta_{i}\sum_{i=1}^{n}\cos^{2}\theta_{i}$, and the equality holds when $\sum_{i=1}^{n-1}\sin2\theta_{i}=0$.

To satisfy this condition, *n* sensors can be divided into n/2 subgroups with two sensors. If the two sensors in each subgroup are placed symmetrically about the *x*-axis, then we have $\sum_{i=1}^{n-1}\sin2\theta_{i}=0$. In addition, the maximum becomes

(20) $$\Upsilon_{2}=\sum_{i=1}^{n}\sin^{2}\theta_{i}\left(n-\sum_{i=1}^{n}\sin^{2}\theta_{i}\right)=-\Upsilon_{22}^{2}+n\Upsilon_{22}=-{\left(\Upsilon_{22}-\frac{n}{2}\right)}^{2}+\frac{n^{2}}{4}$$

where $\Upsilon_{22}=\sum_{i=1}^{n}\sin^{2}\theta_{i}$. Since $\varphi^{\prime }<\pi /2$, then $\sin^{2}\theta_{i}<1/2$ holds, implying $\Upsilon_{22}\in \left[0,n/2\right)$. Take the derivative of $\Upsilon_{2}$ with respect to $\Upsilon_{22}$, yields $\frac{\partial \Upsilon_{2}}{\partial \Upsilon_{22}}=-2\Upsilon_{22}+n>0$, that is $\theta_{i}$ should be as large as possible. The sufficient condition for satisfying the optimal geometries is

(21) $$\left\{\begin{array}{cc} \theta_{i}=\varphi_{i} & i=1,\cdots ,n/2\\ \theta_{i}=-\varphi_{i} & i=n/2+1,\cdots ,n \end{array}\right.$$

the sufficient condition implies that n/2 sensors are placed at each of the two intersections.

If *n* is odd, (12) still holds, that is to say, n−1 sensors can still be grouped in pairs and placed symmetrically about the *x*-axis. Since $\sin^{2}\theta_{i}<1/2$, it yields $\Upsilon_{11}\in \left[0,\left(n-1\right)/2\right)$, then

(22) $$\frac{\partial \Upsilon_{1}}{\partial \Upsilon_{11}}=-2\Upsilon_{11}+\left(n-2\sin^{2}\theta_{n}\right)>1-2\sin^{2}\theta_{n}>0$$

the two sensors in each subgroup should be placed at the two intersections, respectively.

(23) $$\Upsilon_{1}=-\Upsilon_{11}^{2}+n\Upsilon_{11}+\left(n-1-2\Upsilon_{11}\right)\sin^{2}\theta_{n}$$

After the positions of the n−1 sensors are determined, $\Upsilon_{11}$ is a constant, and $n-1-2\Upsilon_{11}>0$, so the *n*th sensor should be placed at any intersection to obtain the maximum of $\Upsilon_{1}$, the sufficient condition for satisfying the optimal geometries is

(24) $$\left\{\begin{array}{cc} \theta_{i}=\varphi_{i} & i=1,\cdots ,(n-1)/2\\ \theta_{i}=-\varphi_{i} & i=(n+1)/2,\cdots ,n-1\\ \theta_{i}=\pm \varphi_{i} & i=n \end{array}\right.$$

### 5.2. The Tangent Angle $\pi /4<\varphi_{t}\leq \pi /3$

In the above Section 5.1, we discuss the optimal geometries according to the parity of the number of sensors and the minimum safety distance. In the following discussions, we can give the same conclusions as long as the maximum feasible angle takes the same range as in Section 5.1.

For the minimum safety distance $r\leq r_{2}$, the maximum feasible angle is $\varphi^{\prime }\geq \pi /2$. If a subgroup contains two sensors and has a separation angle of π/2, then this subgroup is in the optimal geometry. For an even number of sensors, *n*, we can divide them into n/2 such subgroups, then the whole *n* sensors is also in the optimal geometry. On the other hand, if *n* is odd, it follows from the maximum feasible angle that the optimal geometries are the same as that expresses in (17) and (18).

Applying the same idea, if $r>r_{2}$, the maximum feasible angle is less than π/2, then the optimal geometries can be chosen as that in (21) and (24) for even and odd number of sensors, respectively.

### 5.3. The Tangent Angle $\varphi_{t}\leq \pi /4$

If the tangent angle $\varphi_{t}\leq \pi /4$, its maximum feasible angle is less than π/2 regardless of the minimum safety distance. As described in Section 5.2, for even and odd sensors, the optimal geometries are consistent with the sufficient conditions expressed in (21) and (24), respectively.

### 5.4. Arbitrarily Shaped Feasible Deployment Regions

Although the feasible deployment regions discussed above are irregular, they are essentially formed on the basis of a circular region and, therefore, is a regular crescent shape. For irregular regions of arbitrary shape, such as those formed by convex regions and minimum safety distances, it is quite difficult or even impossible to accurately give their shape models and to establish the constrained optimization equations, let alone give their closed-form solutions. We can use such search-based methods as gradient descent algorithm to compute the optimal sensor placement [29].

However, using the maximum feasible angle and separation angles introduced in this paper, we can express the constrained optimization problem more intuitively, and can determine which optimal geometry case it fits in this paper according to the minimum safety distance and the tangent angle.

## 6. Numerical Results

In this section, we present several simulations to demonstrate the relationship between the objective function and the sensor-target geometries. Taking the example shown in Figure 4, suppose that there are two sensors in the localization system, n=2, and the radius of the circular region $F_{C}$ is λ=100 m, the target is located at $S(-200\sqrt{3}/3\;m,0\;m)$ with $\rho =200\sqrt{3}/3$ m. We can obtain that $\varphi_{t}=\pi /3>\pi /4$, $r_{t}=100\sqrt{3}/3$ m, and $r_{1}=\left(100\sqrt{2}+200\right)/\sqrt{6}$ m.

**Figure 7:**
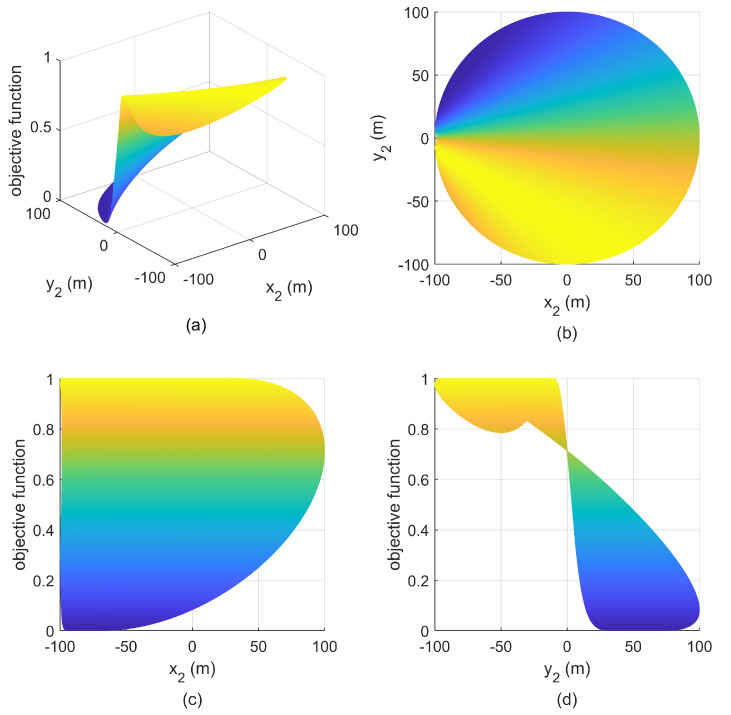
Relationship between objective function and $S_{2}$ with $S_{1}\left(50\sqrt{2}\;m,50\sqrt{2}\;m\right)$ in simulation 1. (**a**) mesh figure; (**b**) X-Y view; (**c**) X-Z view; (**d**) Y-Z view.

**Simulation 1:** We set the minimum safety distance $\rho -\lambda <r=17\;m<r_{5}$, where $r_{5}$ is the length of SC in Figure 3. The sensor $S_{1}$ is located at $\left(50\sqrt{2}\;m,50\sqrt{2}\;m\right)$, and $S_{2}$ can be placed at the whole $F_{C}$, then the relationship between the objective function and the positions of $S_{2}$ is given in Figure 7. We can see that there are infinitely many optimal positions of $S_{2}$, all of which form an optimal separation angle with $S_{1}$, i.e., π/2, and the maximum of the objective function is 1.

**Figure 8:**
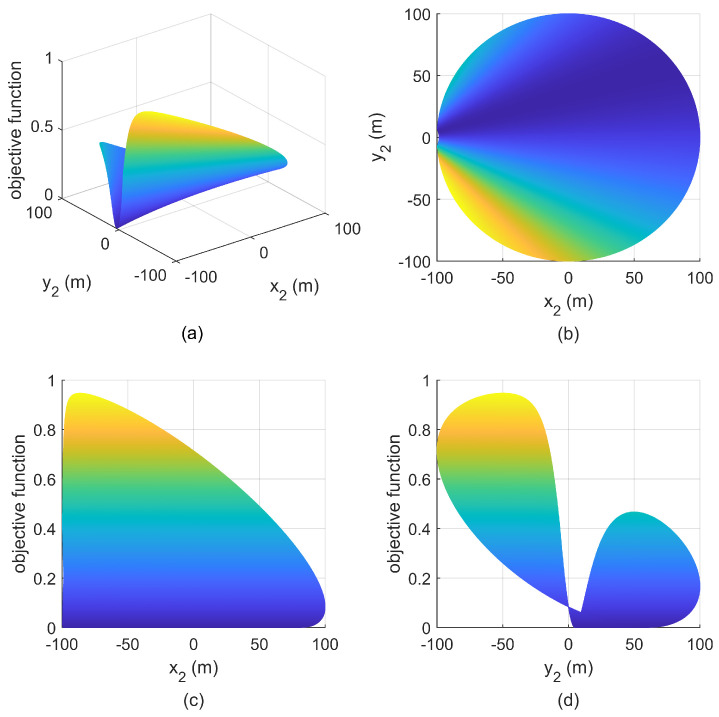
Relationship between objective function and $S_{2}$ with $S_{1}\left(-50\;m,50\;m\right)$ in simulation 1. (**a**) mesh figure; (**b**) X-Y view; (**c**) X-Z view; (**d**) Y-Z view.

Then, we change the position of $S_{1}$ to (−50m,50m), the corresponding simulation result is shown in Figure 8. We can see that the maximum of the objective function at this time is less than the that in Figure 7, which means that the geometry is non-optimal, which also indicates that $S_{1}$ or $S_{2}$ cannot be placed in $F_{C}$; otherwise, it is impossible to achieve the optimal separation angle.

**Figure 9:**
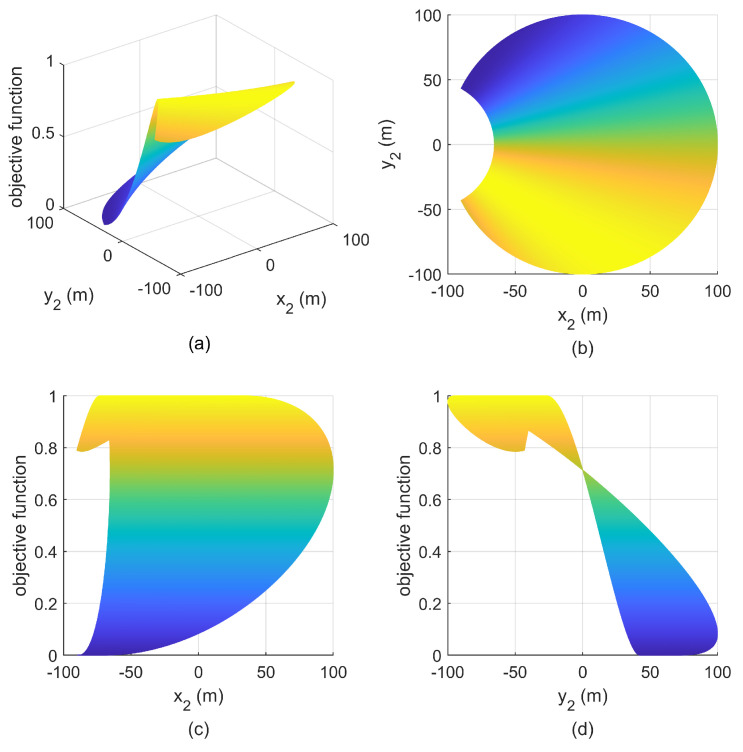
Relationship between objective function and $S_{2}$ with $S_{1}\left(-50\;m,50\;m\right)$ in simulation 2. (**a**) mesh figure; (**b**) X-Y view; (**c**) X-Z view; (**d**) Y-Z view.

**Simulation 2:** Increase the minimum safety distance to $r_{5}<r=50\;m<r_{1}$, and $S_{1}$ is still located at $\left(50\sqrt{2}\;m,50\sqrt{2}\;m\right)$, then it is consistent with that in Figure 4b, the relationship between the objective function and the positions of $S_{2}$ is given in Figure 9. Since the maximum feasible angle is greater than π/2, there are still infinitely many optimal geometries. The difference between Figure 7 and Figure 9 lies in the size of feasible deployment region.

**Figure 10:**
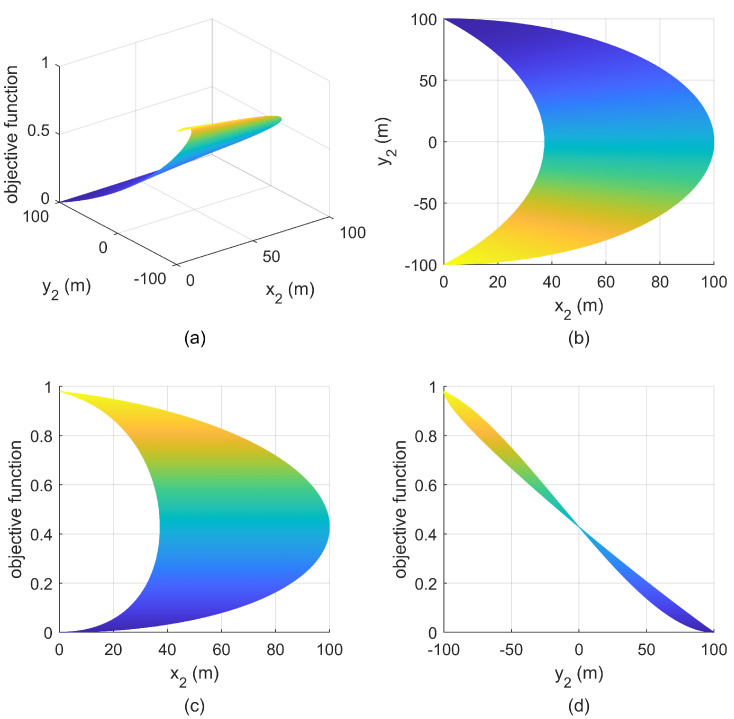
Relationship between objective function and $S_{2}$ with $S_{1}\left(0\;m,100\;m\right)$ in simulation 3. (**a**) mesh figure; (**b**) X-Y view; (**c**) X-Z view; (**d**) Y-Z view.

**Simulation 3:** If the minimum safety distance is greater than $r_{2}$, the maximum feasible angle is $\varphi^{\prime }<\pi /2$. Suppose that the sensor $S_{1}$ is located at (0m,100m), the maximum safety distance is $r=100\sqrt{21}/3$ m, i.e., $S_{1}$ is located at the intersection of $C_{1}$ and $C_{2}$. The relationship between the objective function and the positions of $S_{2}$ is given in Figure 10. We can see that there is only one optimal geometry, that is, $S_{2}$ is located at another intersection.

**Figure 11:**
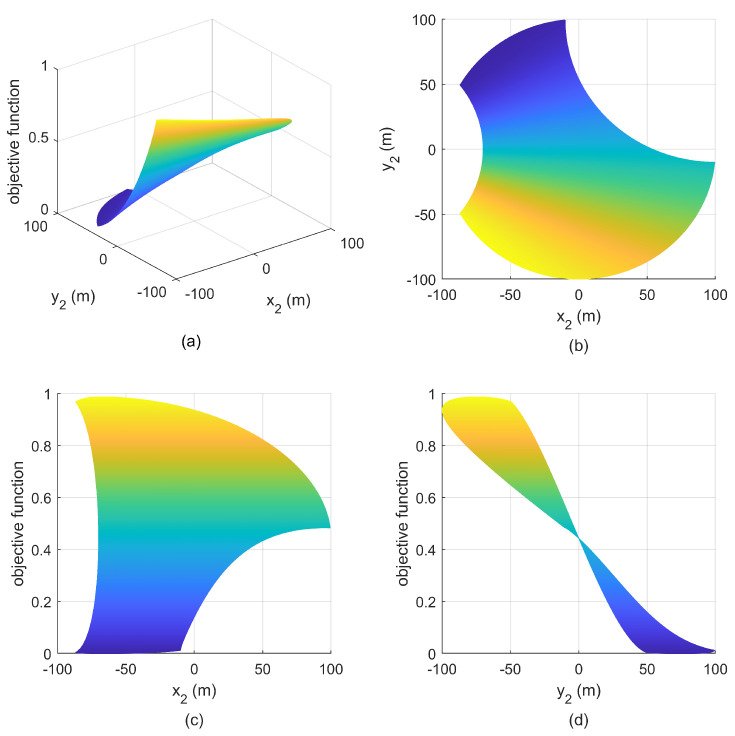
Relationship between objective function and $S_{2}$ with $S_{1}\left(0\;m,100\;m\right)$ in simulation 3. (**a**) mesh figure; (**b**) X-Y view; (**c**) X-Z view; (**d**) Y-Z view.

**Simulation 4:** In this simulation, we will consider the more irregular case, which is formed by three circular regions: $C_{11}\cap C_{22}\cap C_{33}$, where $C_{11}:x^{2}+y^{2}\leq 100^{2}$, $C_{22}:{\left(x-150\right)}^{2}+y^{2}\geq 80^{2}$, $C_{33}:{\left(x+100\right)}^{2}+{\left(y-100\right)}^{2}\geq 110^{2}$. The target is located at S(150m,0m), and $S_{1}$ is located at $\left(50\sqrt{2}\;m,50\sqrt{2}\;m\right)$. The relationship between the objective function and the positions of $S_{2}$ is given in Figure 11.

## 7. Conclusions

This paper mainly focuses on the range-based localization system with the goal of improving target localization accuracy from the geometry optimization perspective, which has been proved in many research works. However, existing optimal sensor geometry strategies usually have the premise that the positions of the sensors are arbitrary, that is, the relative position relationship between the sensors and the target (characterized by the distances, azimuths, and shapes) is arbitrary. This reveals that the sensors need to be placed around the target.

Considering that in practical scenarios, sensor-equipped unmanned platforms may face physical terrain, communication capabilities and security requirements, making them only work in some specific regions. We construct an irregular feasible deployment region defined by a circular initial region and a minimum safety distance, the sensors can be placed inside and on the boundary of this region, while the target is located outside this region. We take the D-optimality criterion as the objective function and the irregular deployment region as the constraints, thus establishing the constrained optimization equations for the geometry optimization problem expressed in terms of the maximum feasible angle and the separation angle. Different maximum feasible angles and different shapes of deployment regions can be obtained by adjusting the minimum safety distance.

We analyzed the optimal geometries with two and three sensors, respectively, and arrive at more complex conclusions than the conventional unconstrained case. The corresponding conclusions can also serve as the basis for the grouping method to provide a reference for an arbitrary number of sensors. Since the mathematical model of the arbitrarily shaped irregular deployment region may not be accurately given, resulting in the inability to solve Equation (7). However, it can be classified into one of the categories discussed in this paper based on the minimum safety distance and maximum feasible angle, and then the conclusions of this paper can be used to provide reference for motion control of unmanned platforms.

The disadvantage is that we mainly consider the circular constrained region, which limits the generality of related theories and methods to a certain extent. However, it is also applicable for certain application scenarios, especially in radar applications. Meanwhile, the method in this paper only considers the maximum feasible angle between the target and the constrained regions, which means that we only need to compare it with the optimal separation angles, eliminating the need for mathematical calculations; therefore, the conclusions in this paper can also be extended to different types of measurements and different shapes of deployment regions.

## Figures and Tables

**Table 1:** Relationship between distances and maximum feasible angle for n=2.

Distances	Tangent Angle	Maximum Feasible Angle	
$\rho \geq \sqrt{2}\lambda$, $r_{t}\geq \lambda$	$\varphi_{t}\leq \pi /4$	$\rho -\lambda <r\leq r_{t}$	$\varphi^{\prime }=2\varphi_{t}\leq \pi /2$
		$r_{t}<r\leq \rho +\lambda$	$\varphi^{\prime }=2\varphi_{i}<2\varphi_{t}\leq \pi /2$
$\lambda <\rho <\sqrt{2}\lambda$, $r_{t}<\lambda$	$\varphi_{t}>\pi /4$	$\rho -\lambda <r\leq r_{t}$	$\varphi^{\prime }=2\varphi_{t}>\pi /2$
		$r_{t}<r\leq r_{1}$	$\varphi^{\prime }=2\varphi_{i}\geq \pi /2$
		$r_{1}<r\leq \rho +\lambda$	$\varphi^{\prime }=2\varphi_{i}<\pi /2$

**Table 2:** Relationship between distances and maximum feasible angle for n=3.

Distances	Tangent Angle	Maximum Feasible Angle	
$\rho \geq \sqrt{2}\lambda$, $r_{t}\geq \lambda$	$\varphi_{t}\leq \pi /4$	$r\leq r_{t}$	$\varphi^{\prime }\leq 2\varphi_{t}\leq \pi /2$
		$r_{t}<r\leq \rho +\lambda$	$\varphi^{\prime }\leq 2\varphi_{i}<2\varphi_{t}\leq \pi /2$
$2\sqrt{3}\lambda /3\leq \rho <\sqrt{2}\lambda$, $\sqrt{3}\lambda /3\leq r_{t}<\lambda$	$\pi /4<\varphi_{t}\leq \pi /3$	$r\leq r_{t}$	$\varphi^{\prime }\leq 2\varphi_{t}\in \left(\pi /2,2\pi /3\right]$
		$r_{t}<r\leq r_{2}$	$\varphi^{\prime }\leq 2\varphi_{i}\in \left(\pi /2,2\varphi_{t}\right)$
		$r>r_{2}$	$\varphi^{\prime }\leq 2\varphi_{i}<\pi /2$
$\rho <2\sqrt{3}\lambda /3$, $r_{t}<\sqrt{3}\lambda /3$	$\varphi_{t}>\pi /3$	$r\leq r_{t}$	$\varphi^{\prime }\leq 2\varphi_{t}\in \left(2\pi /3,\pi \right)$
		$r_{t}<r\leq r_{3}$	$\varphi^{\prime }\leq 2\varphi_{i}\in \left[2\pi /3,2\varphi_{t}\right]$
		$r_{3}<r\leq r_{4}$	$\varphi^{\prime }\leq 2\varphi_{i}\in \left[\pi /2,2\pi /3\right)$
		$r>r_{4}$	$\varphi^{\prime }\leq 2\varphi_{i}<\pi /2$

## Data Availability

Not applicable.

## References

[1] Zhang S., Fan F., Li W., Chu S.C., Pan J.S. (2021). A parallel compact sine cosine algorithm for TDOA localization of wireless sensor network. Telecommun. Syst..

[2] Liang Q., Chu S.C., Yang Q., Liang A., Pan J.S. (2022). Multi-Group Gorilla Troops Optimizer with Multi-Strategies for 3D Node Localization of Wireless Sensor Networks. Sensors.

[3] Huerta M.K., Garcia-Cedeno A., Guillermo J.C., Clotet R. (2021). Wireless sensor networks applied to precision agriculture: A worldwide literature review with emphasis on Latin America. IEEE Geosci. Remote Sens. Mag..

[4] Pannetier B., Dezert J., Moras J., Levy R. (2021). Wireless sensor network for tactical situation assessment. IEEE Sens. J..

[5] Jiang J., Wang G., Ho K.C. (2019). Sensor network-based rigid body localization via semi-definite relaxation using arrival time and doppler measurements. IEEE Trans. Wirel. Commun..

[6] Zheng R., Wang G., Ho K.C. (2021). Accurate semidefinite relaxation method for elliptic localization with unknown transmitter position. IEEE Trans. Wirel. Commun..

[7] Wang Y., Ho K.C. (2018). Unified near-field and far-field localization for AOA and hybrid AOA-TDOA positionings. IEEE Trans. Wirel. Commun..

[8] Naseri M., Amiri H. (2021). A novel bearing-only localization for generalized Gaussian noise. Signal Process..

[9] Saeed N., Nam H. (2016). Cluster based multidimensional scaling for irregular cognitive radio networks localization. IEEE Trans. Signal Process..

[10] Saeed N., Nam H., Al-Naffouri T.Y., Alouini M.S. (2019). A state-of-the-art survey on multidimensional scaling-based localization techniques. IEEE Commun. Surv. Tutor..

[11] Liu J., Guo G. (2021). Vehicle localization during GPS outages with extended Kalman filter and deep learning. IEEE Trans. Instrum. Meas..

[12] Jondhale S.R., Deshpande R.S. (2019). Kalman filtering framework-based real time target tracking in wireless sensor networks using generalized regression neural networks. IEEE Sens. J..

[13] Nardone S., Lindgren A., Gong K. (1984). Fundamental properties and performance of conventional bearings-only target motion analysis. IEEE Trans. Autom. Control.

[14] Gustafsson F., Gunnarsson F. (2005). Mobile positioning using wireless networks: Possibilities and fundamental limitations based on available wireless network measurements. IEEE Signal Process. Mag..

[15] Bishop A.N., Fidan B., Anderson B.D., Doğançay K., Pathirana P.N. (2010). Optimality analysis of sensor-target localization geometries. Automatica.

[16] Xu S., Ou Y., Wu X. (2019). Optimal sensor placement for 3-D time-of-arrival target localization. IEEE Trans. Signal Process..

[17] Sahu N., Wu L., Babu P., MR B.S., Ottersten B. (2022). Optimal sensor placement for source localization: A unified ADMM approach. IEEE Trans. Veh. Technol..

[18] Zhao S., Chen B.M., Lee T.H. (2013). Optimal sensor placement for target localisation and tracking in 2D and 3D. Int. J. Control.

[19] Golihaghighi N., Bighesh M.B. (2021). Using frame theory for optimal placement of new added anchors in location estimation wireless networks. IEEE Trans. Aerosp. Electron. Syst..

[20] Xu S. (2020). Optimal sensor placement for target localization using hybrid RSS, AOA and TOA measurements. IEEE Commun. Lett..

[21] Saeed N., Celik A., Alouini M.S., Al-Naffouri T.Y. (2020). Analysis of 3D localization in underwater optical wireless networks with uncertain anchor positions. Sci. China Inf. Sci..

[22] Li Y., Qi G., Sheng A. (2019). Optimal deployment of vehicles with circular formation for bearings-only multi-target localization. Automatica.

[23] Moreno-Salinas D., Pascoal A.M., Aranda J. (2018). Multiple underwater target positioning with optimally placed acoustic surface sensor networks. Int. J. Distrib. Sens. Netw..

[24] Bo X., Razzaqi A.A., Wang X., Farid G. (2020). Optimal geometric configuration of sensors for received signal strength based cooperative localization of submerged AUVs. Ocean Eng..

[25] Sadeghi M., Behnia F., Amiri R. (2020). Optimal sensor placement for 2-D range-only target localization in constrained sensor geometry. IEEE Trans. Signal Process..

[26] Xu S., Rice M., Rice F. (2021). Optimal TOA-sensor placement for two target localization simultaneously using shared sensors. IEEE Commun. Lett..

[27] Yoo K., Chun J. (2020). Analysis of optimal range sensor placement for tracking a moving target. IEEE Commun. Lett..

[28] Fang X., Yan W., Zhang F., Li J. (2015). Optimal sensor placement for range-based dynamic random localization. IEEE Geosci. Remote Sens. Lett..

[29] Xu S., Doğançay K. (2017). Optimal sensor placement for 3-D angle-of-arrival target localization. IEEE Trans. Aerosp. Electron. Syst..

